# Dissection of quality variation and hierarchical quality evaluation of Evodiae Fructus under multidimensional factors

**DOI:** 10.3389/fpls.2026.1942571

**Published:** 2026-10-28

**Authors:** Yuan-tong Yang, Yi-jing Luo, Yu-qin Luo, Li-ye Pan, Guo-wei Li, Xiang-dong Chen, Dong-mei Sun

**Affiliations:** 1 Guangdong Provincial Key Laboratory of Traditional Chinese Medicine Formula Granule Enterprise, Guangzhou University of Chinese Medicine, Guangzhou, China; 2 Traditional Chinese Medicine Industry and Transformation Discipline, Guangzhou University of Chinese Medicine, Guangzhou, China; 3 Beijing University of Chinese Medicine, Beijing, China; 4 Guangdong Yifang Pharmaceutical Co., Ltd., Guangdong Provincial Key Laboratory of TCM Formula Granule Enterprise, Foshan, China; 5 China Pharmaceutical University, Nanjing, China

**Keywords:** commercial specifications, quality evaluation, alkaloids, random forest, UPLC-Q-Exactive Orbitrap-MS

## Abstract

**Introduction:**

Considerable quality fluctuation and heterogeneity are commonly observed in Evodiae Fructus due to multiple confounding factors. Accordingly, this study performed multi-factor analysis to explore potential key factors associated with its chemical quality, aiming to provide scientific evidence and theoretical support for the quality evaluation of this medicinal material.

**Methods:**

First, for the HPLC-derived multi-component quantitative dataset, the Kruskal-Wallis H test followed by *post-hoc* Dunn’s tests with Benjamini-Hochberg FDR correction was performed, and generalized linear models (GLMs) specified with a Gamma distribution and log-link function together with random forest regression were further constructed to quantify the associations of botanical origins, geographical origins, commercial specifications, and maturity stages with the quality of Evodiae Fructus. Second, for the multi-dimensional UPLC-MS/MS metabolomic profiles, principal component analysis (PCA), orthogonal partial least-squares discriminant analysis (OPLS-DA), Kruskal-Wallis H tests, and fold-change analysis were applied to screen differential metabolites across the predefined comparison groups, with metabolites satisfying VIP > 1, *|log2FC|* > *log2(1.5)*, and unadjusted p < 0.05 retained as differential metabolites. GNPS-based feature-based molecular networking (FBMN) was utilized to visualize the corresponding chemical space. Subsequently, Pearson correlation analysis (|r| > 0.6, *P* < 0.05) and random forest modeling were adopted to filter candidate core markers, which were further confirmed by receiver operating characteristic (ROC) curve analysis (AUC > 0.8). Finally, comparative back-propagation neural network (BPNN) models, with hyperparameters optimized by grid search using 10-fold cross-validation, were established to discriminate different commercial specifications of Evodiae Fructus using both pharmacopoeia-referenced indicators and the selected core biomarkers as input, and their predictive performance was evaluated on an independent test set.

**Results:**

A significant correlation was found between commercial specifications and the differential accumulation of active constituents in Evodiae Fructus. ROC curve validation confirmed that 2-hydroxy-4-methoxy-3-(3’-methylbut-2’-enyl) quinolin-4-one and 1-methyl-[(3E,6Z,9Z,12E)-pentadeca-3,6,9,12-tetraen-1-yl]-4(1H)-quinolone, two alkyl quinolone alkaloids, could be regarded as specific potential biomarkers for quality assessment of Evodiae Fructus. The two candidate markers enable the discrimination of Evodiae Fructus with distinct specifications.

**Conclusions:**

Collectively, this study disentangled the independent and interactive associations between commercial specification and chemical quality of Evodiae Fructus. The candidate markers we obtained enabled BPNN models to effectively discriminate different commercial specifications. These findings provide valuable references for the hierarchical quality assessment of this medicinal herb.

## 1 Introduction

Evodiae Fructus is the dried, nearly ripe fruit of *Evodia rutaecarpa* (Juss). Benth., *Evodia rutaecarpa* (Juss). Benth. var. *officinalis* (Dode) Huang, or *Evodia rutaecarpa* (Juss). Benth. var. *bodinieri* (Dode) Huang, is widely applied in clinical practice for its functions of warming the middle energizer and relieving pain (Lee et al., 2008; Wang et al., 2005). Moreover, Evodiae Fructus exhibits wide geographical distribution and is mainly cultivated and distributed across multiple provinces in China, including Guizhou, Hunan, Jiangxi, Guangxi, Zhejiang and Yunnan. Evodiae Fructus possesses a long medicinal history. Its main chemical constituents include alkaloids, flavonoids, terpenoids and phenylpropanoids, with alkaloids being its primary bioactive constituents (Xia et al., 2023; Wu M.R., et al., 2026). It is a commonly used traditional Chinese medicinal herb with warm interior-dispelling properties, exhibiting extensive clinical applications and reliable therapeutic efficacy. Its related preparations have been widely utilized for the treatment of multiple systemic disorders. In particular, classic gastrointestinal prescriptions containing Evodiae Fructus, including Zuojin Pill, Huatuo Zaizao Pill and Evodiae Fructus Decoction (Wang Y., et al., 2023; Wang N.N., et al., 2026; Wang et al., 2007),exert definitive pharmacological effects such as warming the middle energizer to dispel cold, alleviating pain, soothing the liver and descending qi, and suppressing adverse qi to arr est vomiting. Accordingly, these formulations effectively relieve various gastrointestinal discomforts and improve digestive system disorders.

Under the combined effects of long-term natural evolution, environmental conditions and *ex situ* introduction, Evodiae Fructus has formed abundant variant types. This phenomenon results in mixed original species of commercially available Evodiae Fructus medicinal materials and significant quality disparities among samples from different habitats varies. Furthermore, multiple classification criteria exist (Huang et al., 2018a; Huang et al., 2018b; Zhou, 2016) and the commercial specifications of circulating medicinal materials on the market are inconsistent. This creates an industrial status of “same medicinal material with different labeling standards”, which further leads to unstable quality of medicinal herbs. Nevertheless, commercially available “large-flower Evodiae Fructus “with cracked fruit apices produced in Guangxi still exists on the market. This morphological trait is closely associated with fruit developmental characteristics shaped by its growing environment. Clear classification positioning for this variety is absent in the current standard system. To comply with existing specifications, some merchants mix it into medium-flower batches, or sell it at low prices as inferior medicinal material. Such practices not only hinder the rational utilization of medicinal herb resources but also aggravate fluctuations in medicinal quality.

At present, the quality control of Evodiae Fructus stipulated in the Chinese Pharmacopoeia mainly takes the contents of its characteristic alkaloids and limonin as the core evaluation indices (Chinese Pharmacopoeia Commission, 2025). However, such a single quality control evaluation system cannot comprehensively and objectively reflect the overall quality of Evodiae Fructus medicinal materials. Nevertheless, Evodiae Fructus is affected by multiple interacting factors, resulting in substantial variations in its intrinsic quality. Even samples harvested at the same collection period exhibit considerable quality fluctuations, which seriously hampers the standardization progress of Evodiae Fructus for clinical medication and industrial production (**Figure 1**). Thus, establishing a systematic and comprehensive quality evaluation system and identifying characteristic quality markers is critical for stabilizing Evodiae Fructus quality, uniform efficacy and safe clinical use. Nevertheless, current quality evaluations of Evodiae Fructus mainly focus on individual factors such as geographical origin and commercial specification, while ignoring holistic metabolome-based differential characteristics of medicinal materials (Gao X.J., et al, 2021; Li and Wei, 2015; Zhang et al., 2022; Li et al, 2015; Xu et al., 2017; Wang et al., 2021; Gong, 2008). Accordingly, these studies fail to comprehensively elaborate the intrinsic quality heterogeneity of Evodiae Fructus. In quality evaluation, metabolomics enables holistic metabolic profiling and precise screening of differential metabolites and specific quality markers, overcoming the one-sided limitations of conventional evaluation based on single or multi-component quantification (Xiao et al., 2022; Tian et al., 2022; Luo et al., 2023).

**Figure 1:**
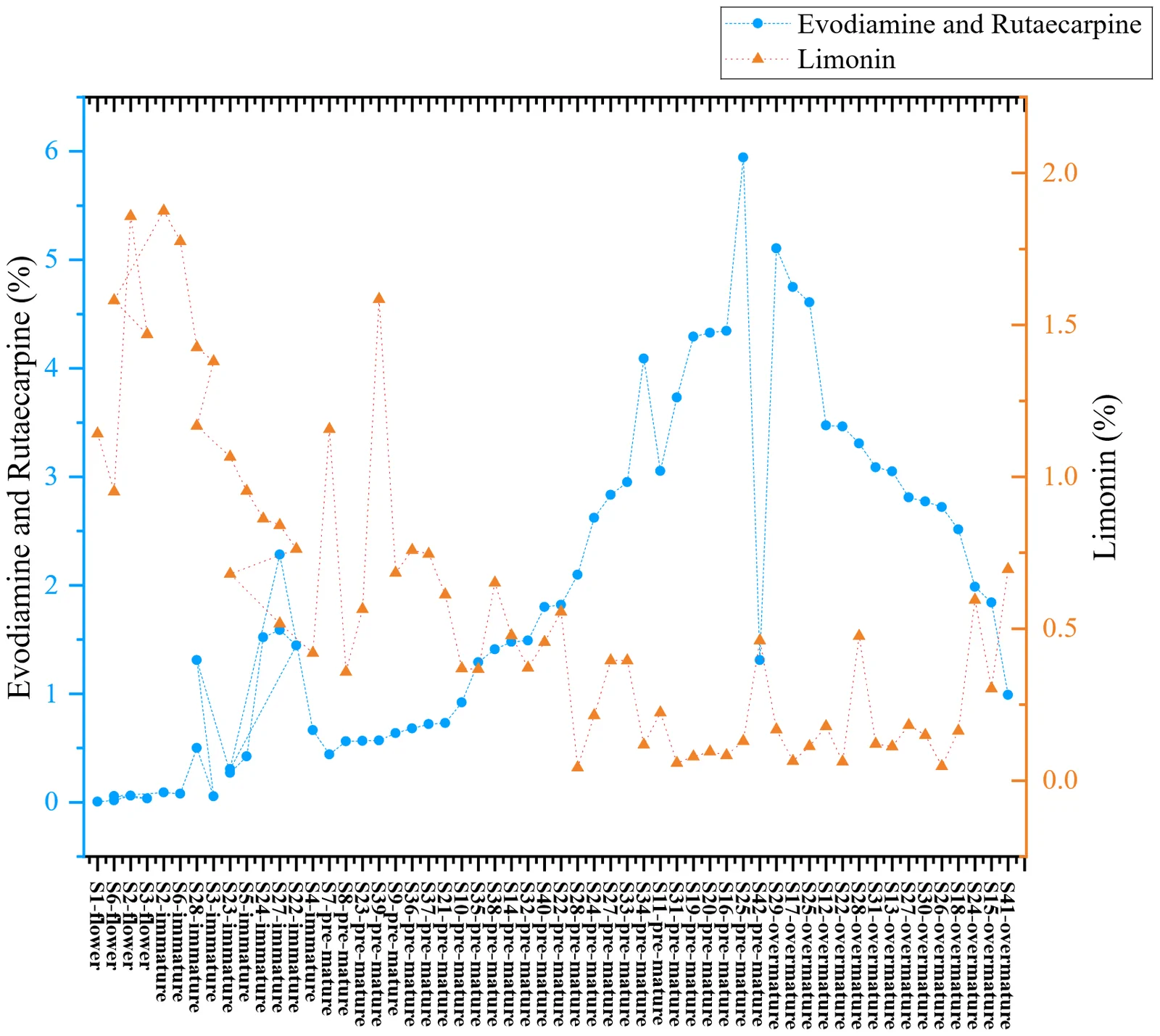
Content trend of total evodiamine and rutaecarpine, and limonin content in Evodiae Fructus samples from different batches.

Therefore, this study aims to establish an analytical strategy based on untargeted metabolomics combined with multivariate statistics and chemometric approaches to systematically dissect the variation patterns of chemical constituents in Evodiae Fructus across four dimensions: geographical origin, maturity stage, botanical origin, and commercial specification. On this basis, variance decomposition and variable-importance evaluation were adopted to preliminarily quantify the relative contributions of these factors to the overall chemical-profile divergence of Evodiae Fructus, and to screen candidate quality markers associated with different groups. Since most previous studies have focused merely on single-factor effects or the quantification of a small number of marker constituents, our findings could provide experimental evidence and data support for the quality evaluation, commercial-specification grading, and standardized production of Evodiae Fructus.

## 2 Materials and methods

### 2.1 Plant material

All samples were collected from herb markets and cultivation bases (**Table 1**). All experimental samples were authenticated by Mr. Liye Pan, Associate Chief Chinese Materia Medica Pharmacist. A total of 42 batches of Evodiae Fructus fruit samples were collected. Several batches contained sub-samples of different maturity stages, yielding 59 experimental samples in total. Among these 42 batches, ten batches (S2, S3, S6, S22, S23, S24, S25, S27, S28, and S31) provided sub-samples from two to four different maturity stages, whereas the remaining 32 batches contributed a single sample each. HPLC multi-index quantitative analysis (including rutaecarpine, evodiamine, limonin) was completed for all samples to investigate inter-batch quality variations. All pre-mature stage samples were selected from the total batch set and subjected to untargeted metabolomics analysis using UPLC-Q-Exactive Orbitrap-HRMS. Four variable dimensions were covered in this analysis, including geographical origin, commercial specification, botanical origin, and maturity stage.

**Table 1:** Collected information and contents of the three markers of the samples (*n* = 2).

Sample no.1	Sample no.2	Botanical origins	Geographical origins	Commercial specifications	Maturity stages
S1	/	*E. rutaecarpa* var. *officinalis*	Hunan	small-flower	flower stage
S6	/	*E. rutaecarpa* var. *officinalis*	Jiangxi	small-flower	flower stage
S2	/	*E. rutaecarpa* var. *officinalis*	Hunan	small-flower	flower stage
S3	/	*E. rutaecarpa* var. *officinalis*	Hunan	small-flower	flower stage
S6	/	*E. rutaecarpa* var. *officinalis*	Jiangxi	small-flower	flower stage
S3	/	*E. rutaecarpa* var. *officinalis*	Hunan	small-flower	immature stage
S6	/	*E. rutaecarpa* var. *officinalis*	Jiangxi	small-flower	immature stage
S2	/	*E. rutaecarpa* var. *officinalis*	Hunan	small-flower	immature stage
S23	/	*E. rutaecarpa* var. *officinalis*	Guangxi	small-flower	immature stage
S5	/	*E. rutaecarpa* var. *officinalis*	Hunan	small-flower	immature stage
S28	YG-S3	*E. rutaecarpa*	Guangxi	small-flower	immature stage
S4	/	*E. rutaecarpa* var. *officinalis*	Hunan	small-flower	immature stage
S22	/	*E. rutaecarpa*	Guangxi	small-flower	immature stage
S27	YG-S2	*E. rutaecarpa*	Guangxi	small-flower	immature stage
S28	PZ-S3	*E. rutaecarpa*	Guangxi	small-flower	immature stage
S24	PZ-S1	*E. rutaecarpa*	Guangxi	small-flower	immature stage
S27	PZ-S2	*E. rutaecarpa*	Guangxi	medium-flower	immature stage
S23	/	*E. rutaecarpa* var. *officinalis*	Guangxi	small-flower	immature stage
S7	XH-S4(SH),JX-S2(SH),	*E. rutaecarpa* var. *officinalis*	Jiangxi	small-flower	pre-mature stage
S8	XH-S5(SH),JX-S4(SH)	*E. rutaecarpa* var. *officinalis*	Jiangxi	small-flower	pre-mature stage
S23	SH-S6,JX-S5(SH)	*E. rutaecarpa* var. *officinalis*	Guangxi	medium-flower	pre-mature stage
S39	XH-S3(SH),JX-S1(SH)	*E. rutaecarpa* var. *officinalis*	Jiangxi	small-flower	pre-mature stage
S9	XH-S6(SH),JX-S3(SH)	*E. rutaecarpa* var. *officinalis*	Jiangxi	small-flower	pre-mature stage
S36	XH-S1(SH),HN-S1(SH),	*E. rutaecarpa* var. *officinalis*	Hunan	small-flower	pre-mature stage
S37	XH-S2(SH),HN-S2(SH)	*E. rutaecarpa* var. *officinalis*	Hunan	small-flower	pre-mature stage
S21	XH-S8(SH),GX-S2(SH)	*E. rutaecarpa* var. *officinalis*	Jiangxi	small-flower	pre-mature stage
S10	XH-S7(SH)	*E. rutaecarpa* var. *officinalis*	Hunan	small-flower	pre-mature stage
S35	SH-S2,GX-S1(SH)	*E. rutaecarpa* var. *officinalis*	Jiangxi	medium-flower	pre-mature stage
S38	SH-S3,HN-S3(SH)	*E. rutaecarpa* var. *officinalis*	Hunan	medium-flower	pre-mature stage
S14	SH-S5,ZH-S3(SH)	*E. rutaecarpa* var. *officinalis*	Guangxi	medium-flower	pre-mature stage
S32	SH-S1,ZH-S1(SH)	*E. rutaecarpa* var. *officinalis*	Jiangxi	medium-flower	pre-mature stage
S40	SH-S4,ZH-S2(SH)	*E. rutaecarpa* var. *officinalis*	Jiangxi	medium-flower	pre-mature stage
S22	WZY-S3,ZH-S1(WZY)	*E. rutaecarpa*	Guangxi	medium-flower	pre-mature stage
S28	JCS-S3,WZY-S6,ZH-S4(WZY),GX-S6(WZY)	*E. rutaecarpa*	Guangxi	medium-flower	pre-mature stage
S24	JCS-S1,WZY-S4,ZH-S2(WZY),HN-S1(WZY)	*E. rutaecarpa*	Guangxi	medium-flower	pre-mature stage
S27	JCS-S2,WZY-S5,ZH-S3(WZY)	*E. rutaecarpa*	Guangxi	medium-flower	pre-mature stage
S33	WZY-S1	*E. rutaecarpa*	Guangxi	medium-flower	pre-mature stage
S34	DH-S2(WZY)	*E. rutaecarpa*	Guangxi	large-flower	pre-mature stage
S11	WZY-S2,GX-S1(WZY)	*E. rutaecarpa*	Guangxi	small-flower	pre-mature stage
S31	HN-S2(WZY)	*E. rutaecarpa*	Hunan	medium-flower	pre-mature stage
S19	DH-S1(WZY),GX-S3(WZY)	*E. rutaecarpa*	Guangxi	large-flower	pre-mature stage
S20	DH-S3(WZY),GX-S4(WZY)	*E. rutaecarpa*	Guangxi	large-flower	pre-mature stage
S16	GX-S2(WZY)	*E. rutaecarpa*	Guangxi	medium-flower	pre-mature stage
S25	DH-S4(WZY),GX-S5(WZY)	*E. rutaecarpa*	Guangxi	large-flower	pre-mature stage
S42	XH-S9(SH)	*E. rutaecarpa* var. *officinalis*	Guizhou	small-flower	pre-mature stage
S29	/	*E. rutaecarpa*	Hunan	large-flower	overmature stage
S17	/	*E. rutaecarpa*	Guangxi	large-flower	overmature stage
S25	/	*E. rutaecarpa*	Guangxi	large-flower	overmature stage
S12	/	*E. rutaecarpa*	Guangxi	medium-flower	overmature stage
S22	/	*E. rutaecarpa*	Guangxi	large-flower	overmature stage
S28	CS-S3	*E. rutaecarpa*	Guangxi	large-flower	overmature stage
S31	/	*E. rutaecarpa*	Hunan	large-flower	overmature stage
S13	/	*E. rutaecarpa*	Guangxi	large-flower	overmature stage
S27	CS-S2	*E. rutaecarpa*	Guangxi	large-flower	overmature stage
S30	/	*E. rutaecarpa*	Hunan	large-flower	overmature stage
S26	/	*E. rutaecarpa* var. *officinalis*	Guangxi	large-flower	overmature stage
S18	/	*E. rutaecarpa* var. *officinalis*	Guangxi	medium-flower	overmature stage
S24	CS-S1	*E. rutaecarpa*	Guangxi	large-flower	overmature stage
S15	/	*E. rutaecarpa*	Guangxi	medium-flower	overmature stage
S41	/	*E. rutaecarpa*	Jiangxi	large-flower	overmature stage

Regarding the statistical treatment of this sampling structure, all statistical analyses in the present study were performed at the level of the experimental sample rather than the batch; that is, each of the 59 experimental samples was treated as an independent observation. This treatment is justified by the fact that sub-samples collected from the same batch at different maturity stages correspond to different commercial specifications of Evodiae Fructus, which are harvested, processed, and traded separately according to maturity stage.

### 2.2 Chemicals and reagents

Evodiamine (Batch No.: 110802-202512, purity 99.8%), rutaecarpine (Batch No.: 110801-202510, purity 99.3%) and limonin (Batch No.: 110800-202308, purity 97.9%) reference standards were purchased from the National Institutes for Food and Drug Control. HPLC-grade methanol and HPLC-grade acetonitrile were obtained from Anke Chemical Co., Ltd. HPLC-grade formic acid and phosphoric acid were supplied by Tianjin Kermel Chemical Reagent Co., Ltd. HPLC-grade tetrahydrofuran was purchased from Kelude Chemical Co., Ltd. Ultrapure water was produced with a Milli-Q Direct system (Merck KGaA).

### 2.3 Standard solution preparation

Accurately weighed evodiamine, rutaecarpine and limonin reference standards were dissolved in methanol, and stock solutions were prepared at concentrations of 80 μg/mL, 50 μg/mL and 100 μg/mL, respectively. These solutions were stored away from light at 4 °C.

### 2.4 Sample solution preparation

Pulverized samples (50-mesh, 0.3 g) were accurately weighed. For HPLC quantification, samples were transferred into 50-mL Erlenmeyer flasks, mixed with 25 mL of 70% ethanol, soaked for 1 h, and subjected to ultrasonic extraction at 300 W and 40 kHz for 40 min. For UPLC-Q-Exactive Orbitrap-HRMS metabolomic analysis, samples were placed in 100-mL Erlenmeyer flasks with 50 mL of 70% methanol and sonicated under identical ultrasonic conditions for 40 min. All extracts were cooled to room temperature, thoroughly mixed, and filtered through a 0.22 μm filter prior to subsequent analysis (Pharmacopoeia of the People’s Republic of China (Vol. 1), 2025 and Xing et al., 2023).

### 2.5 Instruments and chromatographic conditions for content determination

A Waters H-class HPLC system with a quaternary pump, diode-array detector, autosampler, column compartment, and Empower software was used, and chromatographic separation was conducted on an InfinityLab Poroshell 120 SB-C18 column (4.6 mm × 150.0 mm, 2.7 μm, Agilent Technologies, USA).Isocratic elution was performed for 30 min using a mobile phase consisting of acetonitrile-tetrahydrofuran (25:15) mixed with 0.02% phosphoric acid solution at a volume ratio of 35:65. The volume injected was 10 μl. The column temperature was maintained at 30 °C. The detection wavelength was set at 215 nm, and the flow rate was 0.8 mL/min.

### 2.6 Instruments and chromatographic conditions for UPLC-MS/MS analysis

UPLC analysis was carried out on a Thermo Vanquish system (Thermo Fisher Scientific, USA) equipped with a photodiode array detector, column oven, autosampler, and binary solvent delivery system. An YMC Triart C18 column (150 mm × 2.1 mm, 1.9 μm, Guangzhou Techway Biological Technology Co., Ltd., China) was used for chromatographic separation. The column temperatures were set at 30 °C. The injection volume was 1 µL. The mobile phase consisting of 0.1% formic acid-water (A) and acetonitrile (B) was delivered at a flow rate of 0.3 mL/min with linear gradient program as follows: 6% B (0–6 min), 6-20% B (6–14 min), 20-30% B (14–20 min), 30-50% B (20–26 min), 50-70% B (26–35 min), 70-80% B (35–40 min) and 80-95% B (40–45 min).

MS detection was performed using a Thermo UPLC-Q-Exactive Orbitrap mass spectrometer equipped with an electrospray ionization (ESI) source operating in switching ionization mode (Thermo Fisher Scientific, USA).The capillary voltage was 3.30 kV. The capillary temperature was maintained at 350 °C. The mass spectrometer was operated in Full MS/dd-MS² acquisition mode. The resolution was set to 70000 for full-scan MS and 17500 for data-dependent MS². The mass range was 100–1200 m/z, and the normalized collision energy was 30 eV.

### 2.7 Multi-dimensional correlation analysis of quantitative determination data

The four dimensions were encoded and converted into modelable data as follows. For ordinal categorical variables, commercial specifications (small-flower grade = 1, medium-flower grade = 2, large-flower grade = 3), maturity stage (flowering stage = 1, immature stage = 2, pre-mature stage = 3, overmature stage = 4). For nominal categorical variables, botanical origin was coded as: botanical origins (*E. rutaecarpa* = 1, *E. rutaecarpa* var. *officinalis* = 2); geographical origins (Guangxi = 1, Hunan = 2, Jiangxi = 3),and were converted into factor-type encoding for random-forest modeling. Quantitative data were preprocessed via a standardized pipeline. Outliers were detected using the 1.5× interquartile range (IQR) box-plot rule. Of the 59 original samples, only one pre-mature, small-flower sample from Guizhou (S42) was identified as an outlier and excluded, with no other variables or groups affected. All subsequent statistical analyses were performed on the retained 58 samples. Since S42 represented the only Guizhou accession, geographical comparisons were restricted to the three core producing regions (Guangxi, Hunan, and Jiangxi). The filtered dataset was further normalized using the Min–Max method. Sensitivity assessment indicated that exclusion of this single sample (1.7% of the total dataset) exerted negligible impact on overall results. Its removal did not reduce sample coverage for the major geographical groups, and the pre-mature cohort remained the largest among all maturity stages, confirming the robustness of the final dataset and derived conclusions. A standardized data matrix integrating sample attributes and component contents was constructed and saved in Excel/CSV format for subsequent correlation analyses. The Kruskal-Wallis H test (Sherwani et al., 2021; Chan and Walmsley, 1997) and Generalized linear model (GLM) analysis (Mudele et al., 2021; Gao T. et al., 2021) were performed using IBM SPSS 25.0 software, while the random forest regression model (Zhang et al., 2020; Yang et al., 2023; Gu et al., 2023) was constructed using R 4.4.2 software.

The Kruskal-Wallis H test is used to compare data across different dimensions, as the data were showed heterogeneity of variance (Levene’s test, *P* < 0.05). When the omnibus test was significant (P < 0.05), *post-hoc* pairwise comparisons were conducted using Dunn’s test with Benjamini-Hochberg FDR correction (FDR = 0.05) to identify which group pairs differed. Generalized linear models (GLMs) were constructed to assess the associations among categorical variables. According to the distribution characteristics of response variables, models were specified with a Gamma distribution and log-link function and fitted via the maximum-likelihood estimation. Likelihood-ratio *χ²* tests were applied to evaluate model goodness-of-fit, and post-hoc pairwise comparisons were performed with Bonferroni correction. The random forest regression model was trained with ntree = 500 trees, and mtry = max(1, floor(p/3)) variables were sampled for each split, with three distinct random seeds set. Model performance was evaluated exclusively on out-of-bag (OOB) samples and node impurity (IncNodePurity) was also calculated to reflect variable contribution. To reduce sampling perturbations originating from single-model runs, the model was replicated 20 times, and mean values of variable importance were adopted for subsequent analyses.

### 2.8 Multi-dimensional correlation analysis of UPLC-MS/MS data

All data were divided into 6 groups covering four dimensions: different maturity stages, botanical origins, geographical origins, and commercial specifications. Raw data were processed in parallel with two workflows. In the first, MZmine 4.9.0 was used for peak detection, chromatogram deconvolution, alignment, and gap filling; the aligned feature table and MS/MS spectra (.mgf) were exported to GNPS for feature-based molecular networking (FBMN) to visualize chemical relationships among features (Zou et al., 2026). In the second, Compound Discoverer 3.3 was used to generate the quantitation table, with annotations assigned by MS/MS matching against mzCloud, mzVault, and ChemSpider. Differential metabolites were screened exclusively from the Compound Discoverer table; the molecular network served for chemical-space visualization and annotation cross-validation only.

For each group, principal component analysis (PCA) was performed on metabolite peak areas using R 4.4.2 software, and orthogonal partial least squares-discriminant analysis (OPLS-DA) was conducted using SIMCA 14.1 software to screen metabolites with a variable importance in the projection (VIP) value > 1. Subsequently, Kruskal-Wallis H tests (Zhang et al., 2020) were carried out within R 4.4.2 to test for significant differences in metabolite peak-area intensities across groups. Directional fold-change (FC) values were calculated as the ratio of mean peak-area intensities between each comparison group and its corresponding reference group. Metabolites fulfilling *|log2FC|* > *log2(1.5)* and unadjusted P < 0.05 were retained. Metabolites meeting the criteria of VIP > 1, |*log2FC|* > *log2(1.5)*, and *P* < 0.05 were defined as differential metabolites. Heatmaps of differential metabolites in each group were generated using the Bioincloud web - server (https://www.bioinformatics.com.cn/).

### 2.9 Correlation analysis of untargeted metabolomics and quantitative content data

Pearson correlation analysis was used to evaluate associations between differential metabolites and marker contents, and metabolites satisfying |r| > 0.6 and *P* < 0.05 were retained as strongly correlated metabolites. Finally, differential metabolites from the 6 groups were combined. A random forest model was constructed in R 4.4.2 software with differential metabolites as independent variables and Evodiae Fructus content data as the dependent variable. Metabolite importance scores were calculated, and the top 10–15 metabolites with the highest scores were preliminarily screened as candidate core metabolic biomarkers. Receiver operating characteristic (ROC) curve analysis was subsequently performed on these candidates to calculate the area under the curve (AUC) (Zhang et al., 2020; Wu et al., 2026; Wang Z.W., et al., 2023; Du et al., 2026). Biomarkers with AUC values greater than 0.8 exhibited satisfactory discriminatory capacity and were ultimately confirmed as the core metabolic biomarkers. ROC curves were generated to visualize the discriminatory efficiency of the final biomarkers. Finally, comparative back-propagation neural network (BPNN) models were established to discriminate different commercial specifications of Evodiae Fructus, using both pharmacopoeia-referenced indicators and the selected core candidate markers as input (Carracedo-Reboredo et al., 2021). The BPNN classification model samples were divided into a training set and a test set using stratified random sampling at a ratio of 7:3 to ensure that the proportions of each specification category remained consistent. The network hyperparameters were optimized via grid search using 10-fold cross-validation on the training set: the number of hidden layer nodes (size) was set to 8, 16, or 32; the weight decay coefficient (decay) was set to 0.001,0.01, or 0.1; and the maximum number of iterations (maxit) was set to 1000; the cross-validation accuracy (Accuracy) was used as the criterion for model selection. The optimal hyperparameter combination was then used to train the final model, and its predictive performance was evaluated on an independent test set, with the overall accuracy as well as the precision, recall, and F1 score for each category reported.

## 3 Results and discussion

### 3.1 Multi-dimensional analysis to explore the key factors affecting the quality of Evodiae Fructus

#### 3.1.1 Analysis of influence degree based on single dimension

##### 3.1.1.1 Kruskal-Wallis H test

Statistical analysis was performed using SPSS 25.0 software. First, normality and homogeneity of variance for the total contents of evodiamine, rutaecarpine and limonin in each Evodiae Fructus group were evaluated using the Kolmogorov-Smirnov (K-S) test (n ≥ 50).As shown in **Tables 2** and **3**, data from all groups violated the assumptions of normality and homogeneity of variance (*P* < 0.05). Accordingly, the non-parametric Kruskal-Wallis H test was used to compare overall distributional differences among multiple independent groups. The Kruskal-Wallis H test revealed that all four influencing factors exerted significant effects on the contents of marker compounds, after which *post-hoc* pairwise comparisons were performed between groups (**Table 4**). The combined contents of evodiamine and rutaecarpine followed an overall trend: samples from Guangxi > Jiangxi and Hunan; *E. rutaecarpa* > *E. rutaecarpa* var. *officinalis*; large and medium-sized flowers > small flowers; pre-mature and overmature samples > immature samples. By contrast, limonin levels showed no obvious variation among different producing regions, and its changing trends across origins, fruit specifications and maturity stages were opposite to those of the combined contents of evodiamine and rutaecarpine.

**Table 2:** Homogeneity test of variances between influencing factors and component contents.

Contents (%)	Influencing factors	Levene statistic	Sig.
Evodiamine and Rutaecarpine	botanical origins	4.624	0.036
	geographical origins	2.239	0.116
	commercial specifications	2.650	0.080
	maturity stages	5.203	0.003
Limonin	botanical origins	8.544	0.005
	geographical origins	3.489	0.037
	commercial specifications	14.922	0.6E-5
	maturity stages	3.367	0.025

**Table 3:** Normality test of component contents.

Contents (%)	K-S value	F	Sig.
Evodiamine and Rutaecarpine	0.135	59	0.009
Limonin	0.141	62	0.005

**Table 4:** Kruskal-Wallis test with *post-hoc* multiple pairwise comparisons.

Influencing factors	Degree of influence	Evodiamine and rutaecarpine contents%	Limonin contents%
geographical origins	Guangxi	2.950^a^	0.303^a^
	Jiangxi	0.570^b^	0.680^a^
	Hunan	0.720^b^	0.651^a^
botanical origins	*E. rutaecarpa*	2.950^a^	0.198^a^
	*E. rutaecarpa* var. *officinalis*	0.570^b^	0.684^b^
commercial specifications	small-flower	0.470^a^	1.002^a^
	medium-flower	1.819^b^	0.456^b^
	large-flower	3.068^b^	0.125^b^
maturity stages	flower stage	0.034^a^	1.469^a^
	immature stage	0.462^a^	1.009^a^
	pre-mature stage	1.800^b^	0.395^b^
	overmature stage	3.049^b^	0.164^b^

Different lowercase letters within the same row indicate significant differences at P<0.05; values sharing any common letter are not significantly different. Label ‘ab’ means no significant difference with groups marked ‘a’ or ‘b’.

##### 3.1.1.2 Random forest regression model

Variable importance analysis was further performed to quantify the contribution weight of each influencing dimension to the accumulation of target active constituents, and the corresponding results are illustrated in **Figure 2**.The model achieved an out - of - bag (OOB) explained variance of 55.80 %, with an out - of - bag mean squared error (OOB - MSE) equal to 0.034 for the combined evodiamine and rutaecarpine contents prediction and an out - of - bag (OOB) explained variance of 71.16%, with an out - of - bag mean squared error (OOB - MSE) equal to 0.018 for limonin content prediction. Fruit maturity stage (IncNodePurity = 1.050 ± 0.037) and commercial specification (IncNodePurity = 1.020 ± 0.042) were the top - ranked factors for limonin content, whereas commercial specification (IncNodePurity = 0.914 ± 0.049) governed the combined evodiamine - rutaecarpine content. These results demonstrate that commercial specification is a critical source of quality variation among Evodiae Fructus samples.

**Figure 2:**
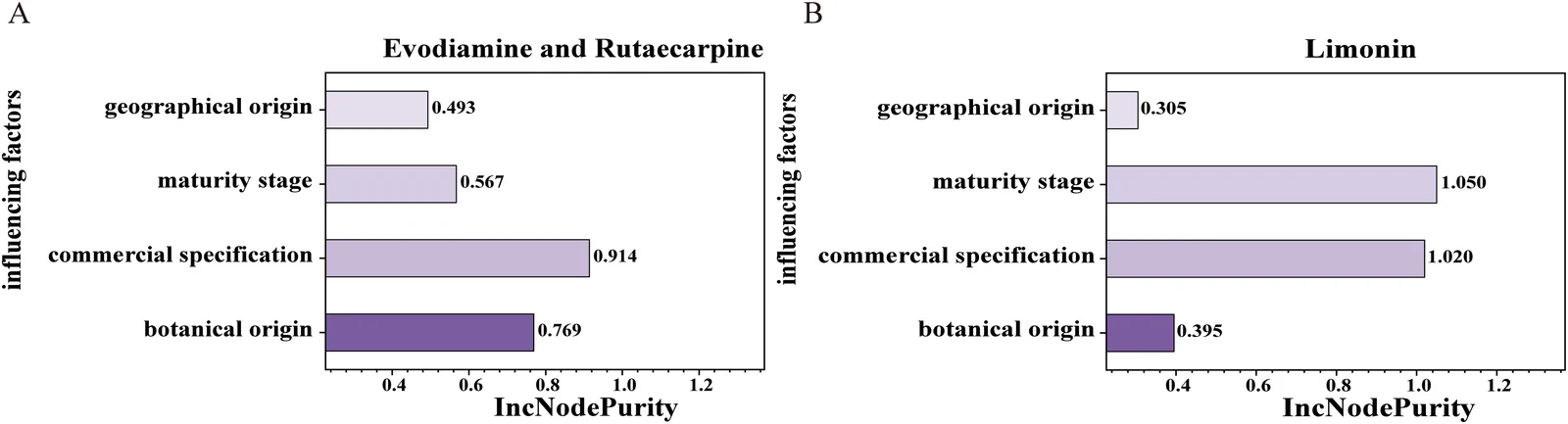
Variable importance of random forest model for total evodiamine and rutaecarpine and limonin.

#### 3.1.2 Generalized linear model analysis with Gamma distribution and log - link function

Cross - tabulation and association analyses revealed significant confounding effects among the four categorical variables (**Supplementary Table S1**). To complement and verify the variable importance ranking derived from random forest modeling, generalized linear models (Gamma distribution with log-link function) were further applied to evaluate the main effects, pairwise interaction effects, and stratified effects of each predictor on target metabolite contents.

For the combined contents of evodiamine and rutaecarpine (**Tables 5**, **6**), maturity stage exhibited the dominant main effect (Wald *χ²* = 119.182). All second-order interaction terms showed no statistical significance (*P* ≥ 0.561), and several interaction terms were non-estimable due to empty cross-classification cells. Given the potential confounding between commercial specification and maturity stage (Wald *χ²* = 1.157, *P* = 0.561), further stratified analysis was performed to clarify the net specification effect within each maturity subgroup. In the pre-mature stage, significant specification-dependent differences were observed: small-flower samples exhibited markedly lower alkaloid accumulation than large-flower samples (Exp(B) = 0.250, 95% CI: 0.150~0.415, *P* < 0.001), and medium-flower samples also presented lower content relative to large-flower samples (Exp(B) = 0.482, 95% CI: 0.302~0.769, *P* = 0.002).

**Table 5:** Type-III Wald test for predictors of combined evodiamine - rutaecarpine content based on a Gamma generalized linear model (log link, n=59).

Source	Wald *χ²*	df	*P*
maturity stages	119.182	3	0.00E0
commercial specifications	13.194	2	0.001
geographical origins	6.108	2	0.047
botanical origins	0.951	1	0.607
geographical origins * botanical origins	0.268	1	0.605
geographical origins * commercial specifications	0.204	2	0.903
geographical origins * maturity stages	0.929	3	0.818
commercial specifications * maturity stages	1.157	2	0.561

**Table 6:** Stratified analysis: effects of commercial specification on combined evodiamine - rutaecarpine content across maturity subgroups (Gamma GLM, log link).

Maturity stages	Commercial specifications	Wald *χ²*	Exp(B)	95%CI	*P*
immature stage	small-flower vs. medium-flower	1.861	0.268	0.040~1.777	0.172
pre-mature stage	small-flower vs. large-flower	28.678	0.250	0.150~0.415	8.54E-8
	medium-flower vs. large-flower	9.394	0.482	0.302~0.769	0.002
overmature stage	medium-flower vs. large-flower	0.505	0.826	0.488~1.398	0.477

For limonin content (**Tables 7**, **8**), commercial specification constituted the strongest influencing factor (Wald *χ²* = 27.82, *P* < 0.001). A significant interaction between commercial specification and geographic origin was detected (Wald *χ²* = 8.98, *P* = 0.011), indicating that the magnitude of the specification effect varied across producing areas. Nevertheless, the effect direction remained consistent among all three origins: small-flower samples always yielded significantly higher limonoid content than large-flower samples (Guangxi: Exp(B) = 4.625; Hunan: Exp(B) = 7.705; Jiangxi: Exp(B) = 4.493; all *P* < 0.001). The effect intensity of commercial specification exhibited distinct geographic heterogeneity. The Hunan population showed the strongest specification gradient, with medium-flower samples also possessing significantly higher limonoid content than large-flower samples (Exp(B) = 4.375, *P* < 0.001). By contrast, no significant differences were detected between medium- and large-flower samples in Guangxi (Exp(B) = 1.698, *P* = 0.080) and Jiangxi populations (Exp(B) = 2.059, *P* = 0.092).

**Table 7:** Type-III Wald test for predictors of limonin content based on a Gamma generalized linear model (log link, n=59).

Source	Wald *χ²*	df	*P*
commercial specifications	27.815	2	9.12E-7
maturity stages	6.479	3	0.091
geographical origins	4.541	2	0.0103
botanical origins	4.220	1	0.040
geographical origins * botanical origins	0.204	1	0.652
geographical origins * commercial specifications	8.980	2	0.011
geographical origins * maturity stages	5.635	3	0.131
commercial specifications * maturity stages	1.600	2	0.449

**Table 8:** Stratified analysis: effects of geographical origins on limonin content across maturity subgroups (Gamma GLM, log link).

Geographical origins	Commercial specifications	Wald *χ²*	Exp(B)	95%CI	*P*
Guangxi	small-flower vs. large-flower	17.777	4.625	2.269~9.425	2.50E-5
	medium-flower vs. large-flower	3.057	1.698	0.938~3.072	8.04E-2
Hunan	small-flower vs. large-flower	69.556	7.705	4.768~12.450	1.11E-16
	medium-flower vs. large-flower	15.575	4.375	2.102~9.106	7.90E-5
Jiangxi	small-flower vs. large-flower	17.044	4.493	2.202~9.170	3.70E-5
	medium-flower vs. large-flower	2.842	2.059	0.889~4.766	0.092

The combined contents of evodiamine and rutaecarpine increased mainly with the advancement of maturity stages and exhibited significant independent associations with commercial specification, geographic origin, and botanical origin. Stratified analysis further revealed that the specification-dependent variation of these alkaloids was predominantly pronounced in near-mature fruits, with distinct differences observed between small-flower and other commercial grades. In contrast, limonin accumulation was primarily governed by commercial specification, and the magnitude of such specification effects was significantly modulated by geographic origin. Collectively, the divergent responses of combined evodiamine - rutaecarpine and limonin components to commercial grading indicated that targeted optimization of harvesting schedules and commercial classification standards should be implemented according to the specific chemical profiles of Evodiae Fructus ingredients.

### 3.2 Screening of differential metabolites of Evodiae Fructus based on multi-dimensional analysis

To comprehensively examine metabolic variation across the six comparative dimensions, PCA and OPLS-DA analyses were performed on a merged dataset comprising 61 samples and 690 metabolite features (**Figure 3**). The first two principal components of the PCA model collectively explained 94.5% of the total variation (R²X = 0.945, Q² = 0.889), indicating that the model possesses strong explanatory and predictive power. The OPLS-DA model (R²X = 0.895, R²Y = 0.992, Q² = 0.989) effectively discriminated between the six comparative groups: samples from different geographical origins of *E. rutaecarpa* and *E. rutaecarpa* var. *officinalis* clustered into separate clusters; the commercial - specifications group and the botanical-origins group of *E. rutaecarpa* were located in the central region of the score plot with partial overlap, suggesting that the metabolic differences among these dimensions are relatively subtle. The separate PCA and OPLS-DA models for each comparative dimension, along with their corresponding model parameters, are presented in **Supplementary Figure 1**. The final number of differentially regulated metabolites was visualized using a Venn diagram (**Supplementary Figure 2**). The molecular network analysis diagram based on GNPS features (GNPS-FBMN) is shown in **Supplementary Figures 3**-**8**. All differential metabolites were combined to generate a clustering heatmap to illustrate the alterations in these metabolites among various groups (**Figure 4**).The detailed information of all differential metabolites is summarized in **Supplementary Table 2**.

**Figure 3:**
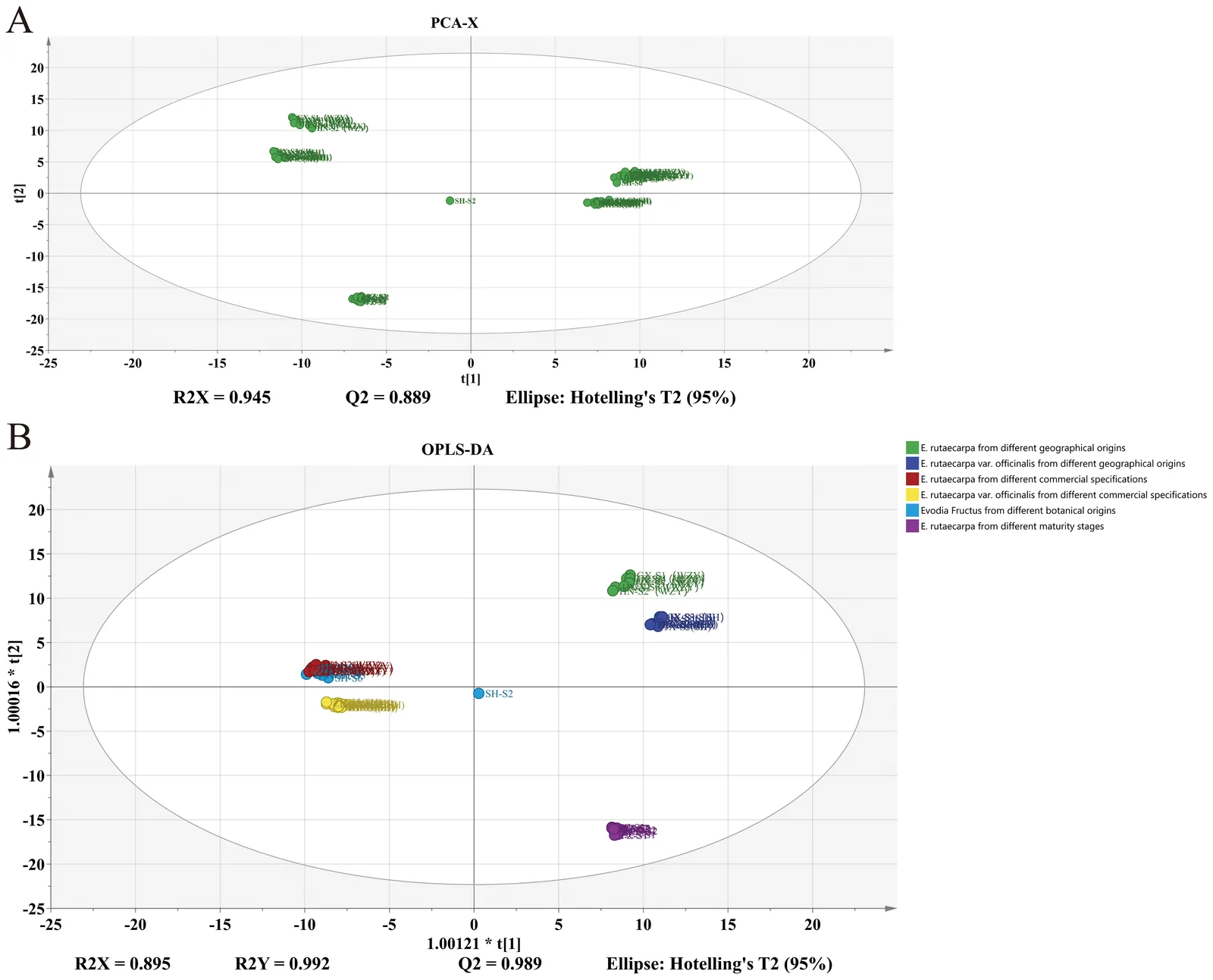
Integrated multivariate analysis of the non-targeted metabolome across all six comparative dimensions of Evodiae Fructus (n = 61 samples, 690 metabolomic features).Some samples were collected repeatedly across multiple groups. **(A)** Unsupervised principal component analysis (PCA) score plot showing the overall metabolic variation among all samples; each point represents one sample, and the ellipse denotes the Hotelling’s T² (95%) confidence region. **(B)** Supervised orthogonal projections to latent structures discriminant analysis (OPLS-DA) score plot with the six comparative groups color-coded: *E. rutaecarpa* from different geographical origins(green), *E. rutaecarpa* var. *officinalis* from different geographical origins (navy blue), *E. rutaecarpa* from different commercial specifications(red), *E. rutaecarpa* var. *officinalis* from different commercial specifications(yellow), Evodiae Fructus from different botanical origins (wathet blue), and *E. rutaecarpa* from different maturity stages(purple).

**Figure 4:**
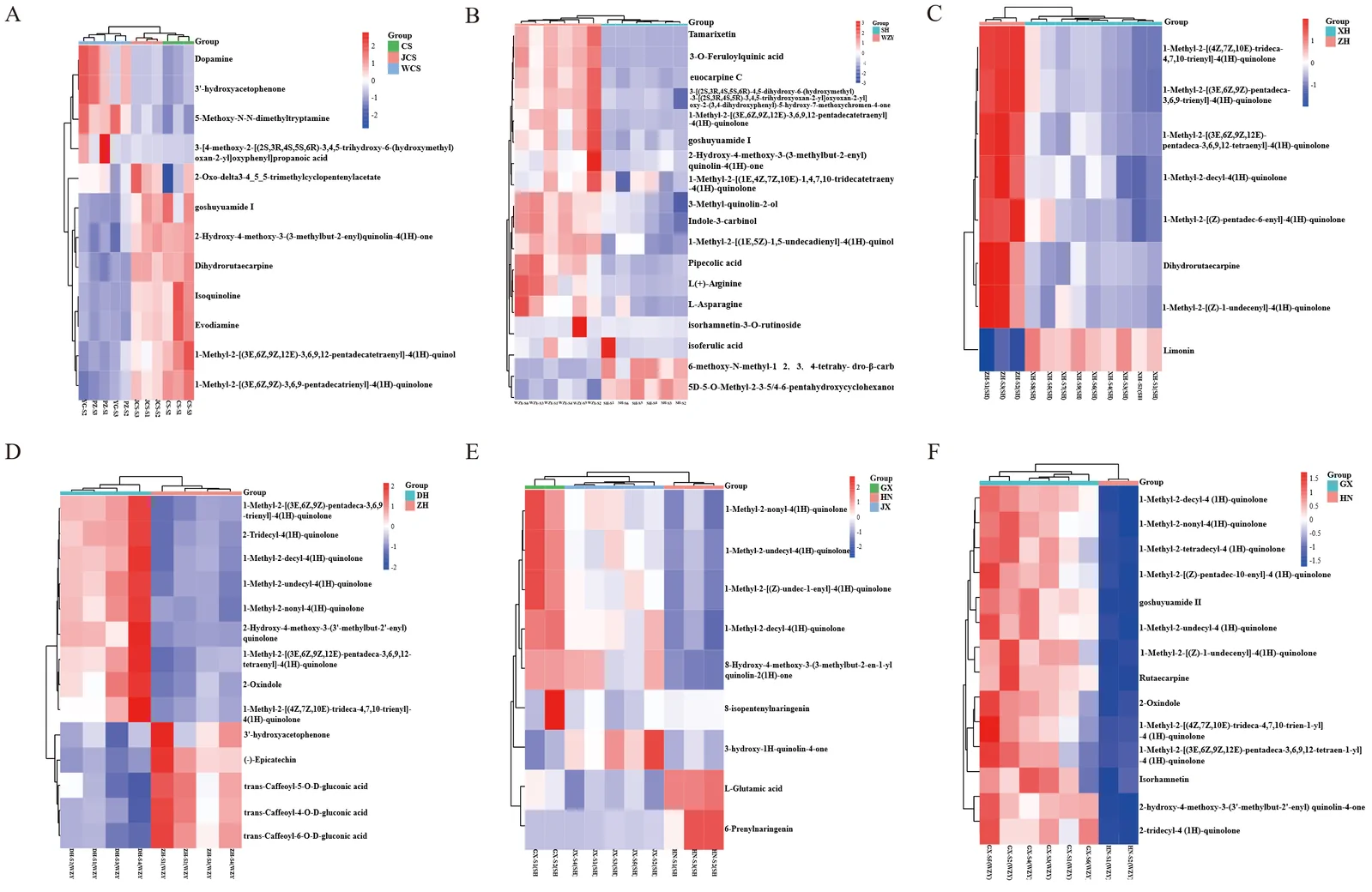
Heatmap analysis of differentially expressed metabolites based on all six comparative dimensions of Evodiae Fructus. **(A)** *E. rutaecarpa* from different maturity stages. **(B)** Evodiae Fructus from different botanical origins. **(C)** *E. rutaecarpa* var. *officinalis* from different commercial specifications. **(D)** *E. rutaecarpa* from different commercial specifications. **(E)** *E. rutaecarpa* var. *officinalis* from different geographical origins. **(F)** *E. rutaecarpa* from different geographical origins.

Analysis based on diverse maturity stages: The results revealed that characteristic alkaloids, including goshuyuamide I and evodiamine, as well as core bioactive constituents such as quinoline alkaloids and isoquercitrin, were significantly upregulated in pre-mature and overmature *E. rutaecarpa* samples. In contrast, amide compounds, including dopamine, 5-methoxy-N-N-dimethyltryptamine, and 3’-hydroxyacetophenone, were markedly enriched in immature fruits. In addition, obvious individual fluctuations in the relative abundance of differential metabolites were observed among different samples within the same maturity group.

Analysis based on diverse botanical origins: The results revealed that typical indole and quinoline alkaloids, including goshuyuamide I and 1-methyl-2-[(1E,5Z)-undeca-1,5-dien-1-yl]-4(1H)-quinolone, exhibited intense red signals corresponding to high relative abundance in *E. rutaecarpa* samples. In comparison, *E. rutaecarpa* var. *officinalis* samples were predominantly enriched with carboline alkaloids (e.g., 6-methoxy-N-methyl-1,2,3,4-tetrahydro-β-carboline) and phenylpropanoids such as isoferulic acid. Moreover, several exclusive differential metabolite enrichment characteristics were specifically observed in certain *E. rutaecarpa* var. *officinalis* samples.

Analysis based on diverse commercial specifications: The medicinal specifications of *E. rutaecarpa* var. *officinalis* are mainly medium-flower and small-flower grades. Medium-flower samples accumulated abundant quinolone alkaloids, while small-flower samples possessed higher limonin contents. In contrast, *E. rutaecarpa* was mainly distributed as large-flower and medium-flower grades. large-flower samples were enriched with quinolone alkaloids, whereas medium-flower samples exhibited prominent accumulation of phenolic acids and flavonoids. Overall, distinct metabolic profiles were observed between the two botanical origins.

Analysis based on diverse geographical origins: For *E. rutaecarpa* var. *officinalis* samples from different geographical origins, collected from Guangxi were rich in quinolone alkaloids, such as 1-methyl-2-nonyl-4(1H)-quinolone. Samples from Hunan were characterized by flavonoids and amino acid metabolites, with specific upregulation of 6-isopentenylnaringenin and L-glutamate. Samples from Jiangxi, 3-hydroxy-1H-quinolin-4-one was significantly elevated, and several other alkaloid constituents also presented varying degrees of upregulation in partial samples. Regarding *E. rutaecarpa* samples from different producing areas, Guangxi samples exhibited prominent accumulation of quinolone alkaloids, while Hunan samples showed specifically increased levels of fatty acid metabolites. In addition, locally high-abundance light-red enrichment bands of metabolites were observed within the Guangxi sample group. Collectively, significant batch-to-batch metabolic variations existed among Evodiae Fructus samples from the same geographical origin.

### 3.3 Screening of core metabolic markers by machine learning combined with correlation analysis

#### 3.3.1 Correlation analysis

Pearson correlation analysis between differential metabolites and the contents of multi-index bioactive components was performed in R software to calculate the correlation coefficient r and significance p-value. Metabolites satisfying the thresholds of |r|>0.6 and p<0.05 were screened out (**Figure 5**). Correlation analysis revealed that indole alkaloids(goshuyuamide I, 2-Oxindole, Dihydrorutaecarpine, et al), quinoline alkaloids(1-Methyl-2-decy -4(1H)-quinoloneand, 2-Tridecyl-4(1H)-quinolone, 1-Methyl-2-undecyl-4(1H)-quinolone, et al)and flavonoid metabolites(Isorhamnetin) were significantly positively correlated with the total content of evodine and rutaecarpine, while certain alkaloids(5-Methoxy-N-N-dimethyltryptamine, 5D-5-O-Methyl-2-3-5/4-6-pentahydroxycyclohexanone), limonin and phenylpropanoid(trans Caffeoyl-5-O-D-gluconic acid,(-)-Epicatechin, et al) metabolites showed significant negative correlations with this total index.5-Methoxy-N-N-dimethyltryptamine, trans-Caffeoyl-4-O-D-gluconic acid and (-)-Epicatechin were significantly positively correlated with the total limonoin content, whereas alkaloid metabolites showed significant negative correlations with limonin levels.

**Figure 5:**
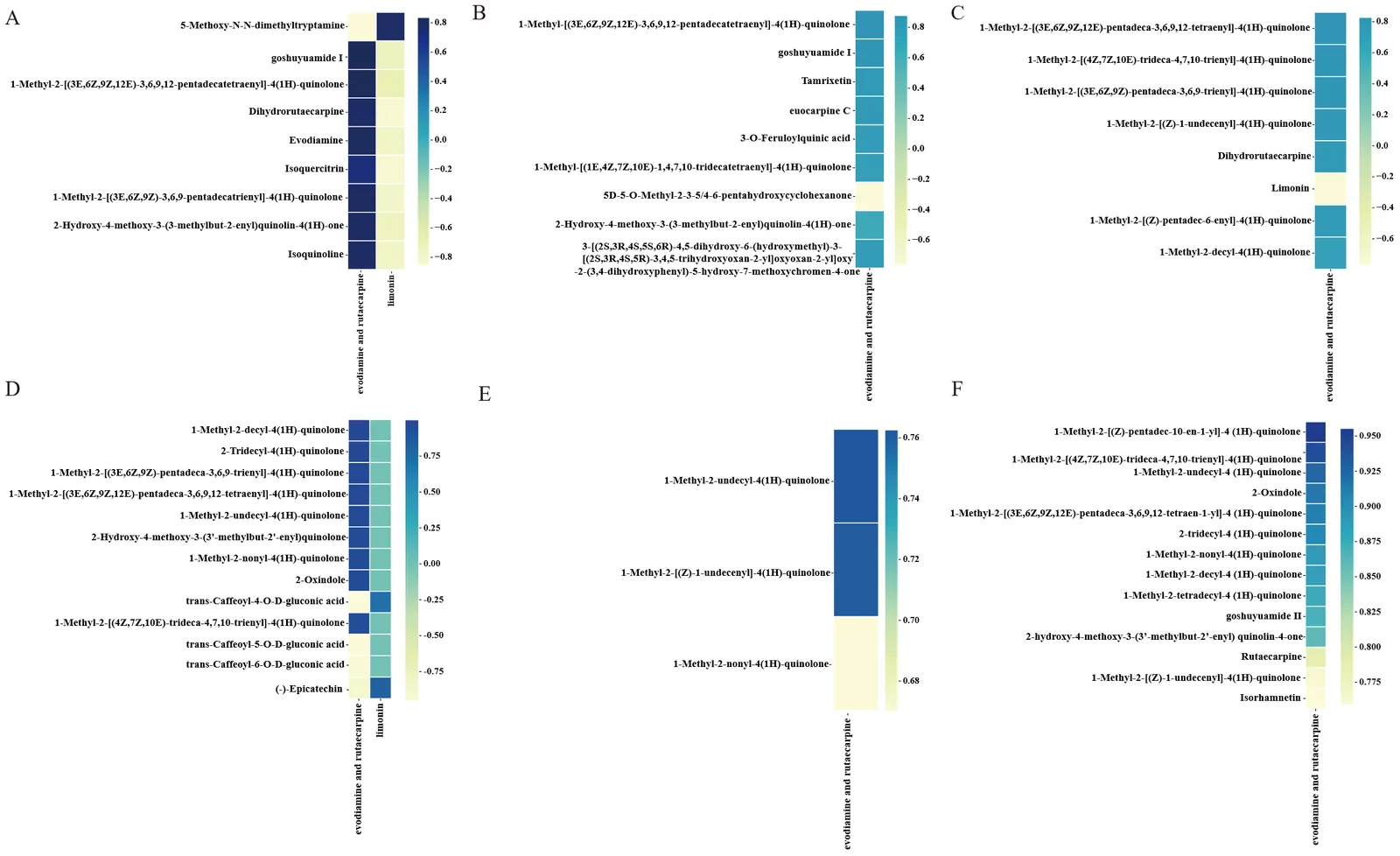
Core metabolites screened from different dimensions. **(A)** diverse maturity stages, **(B)** diverse botanical origins, **(C)** diverse commercial specifications (*E. rutaecarpa* var. *officinalis*), **(D)** diverse commercial specifications (*E. rutaecarpa*), **(E)** diverse geographical origins (*E. rutaecarpa* var. *officinalis*), **(F)** diverse geographical origins (*E. rutaecarpa*).

#### 3.3.2 Random forest machine learning analysis

All core differential metabolites preliminarily screened in section 3.3.1 were combined and deduplicated. A random forest classification model was constructed with the integrated metabolites as independent variables and multi-index quantitative data as dependent variables. The top 15 metabolites ranked by variable importance score were selected as candidate characteristic markers, and all metabolites were ranked by their AUC values to obtain the top 15 metabolites (**Figures 6A, B**). ROC curve analysis was performed on each candidate metabolite individually to evaluate their discriminative capacity. Comprehensive AUC results indicated that 2-hydroxy-4-methoxy-3-(3’-methylbut-2’-enyl) quinolin-4-one and 1-Methyl-[(3E,6Z,9Z,12E)-pentadeca-3,6,9,12-tetraen-1-yl]-4(1H)-quinolone could serve as potential biomarkers of Evodiae Fructus, and the combined application of the two candidate markers could further improve the discrimination accuracy of Evodiae Fructus samples from different commercial specifications (**Figure 6C**).

**Figure 6:**
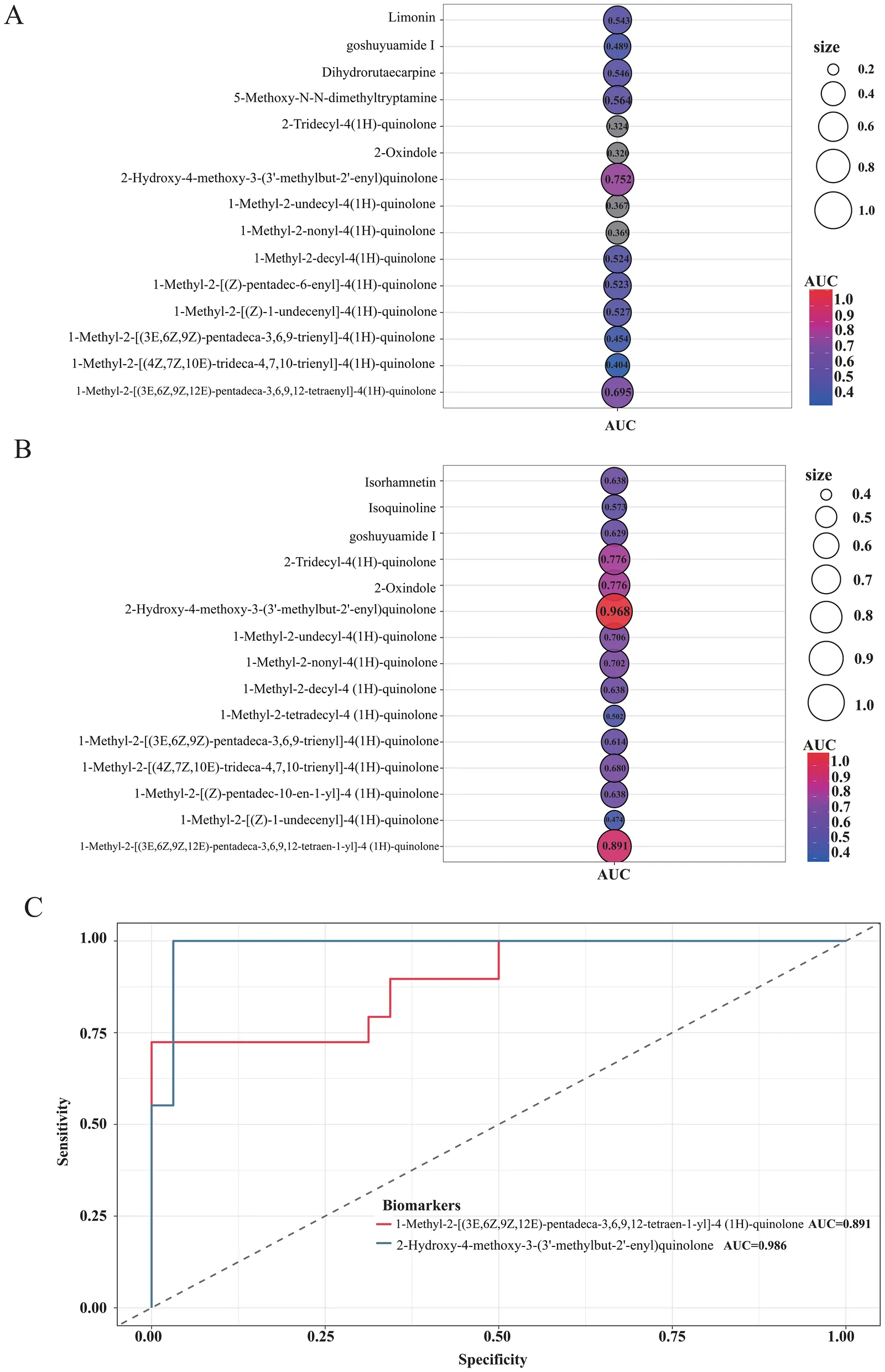
AUC ranking results and ROC curves. **(A)** Ranking of metabolites affecting the combined contents of evodiamine and rutaecarpine by AUC value and variable importance. **(B)** Ranking of metabolites affecting limonin content by AUC value and variable importance. **(C)** Screening of core metabolic markers affecting the combined contents of evodiamine and rutaecarpine based on ROC curves.

#### 3.3.3 BPNN machine learning analysis

Collectively, the random forest model, generalized linear model, and clustering heatmap consistently demonstrated that commercial specification exerts a significant independent effect on the quality variation of Evodiae Fructus materials, indicating that commercial specification should be regarded as a critical basis for the quality control of this medicinal herb. A backpropagation neural network (BPNN) model was further applied to verify whether the two candidate markers screened in this study could effectively distinguish samples with different commercial specifications (**Figure 7**). Cross-validation effectively avoided bias in the absence of real test set. Accuracy, recall, precision, F1 and predicted probability for each test set were employed for model evaluation (**Supplementary Table 3**). Models were constructed based on pharmacopoeia marker compounds and the two candidate markers, respectively. The results revealed that the two candidate markers correctly classified all seven reserved test samples.

**Figure 7:**
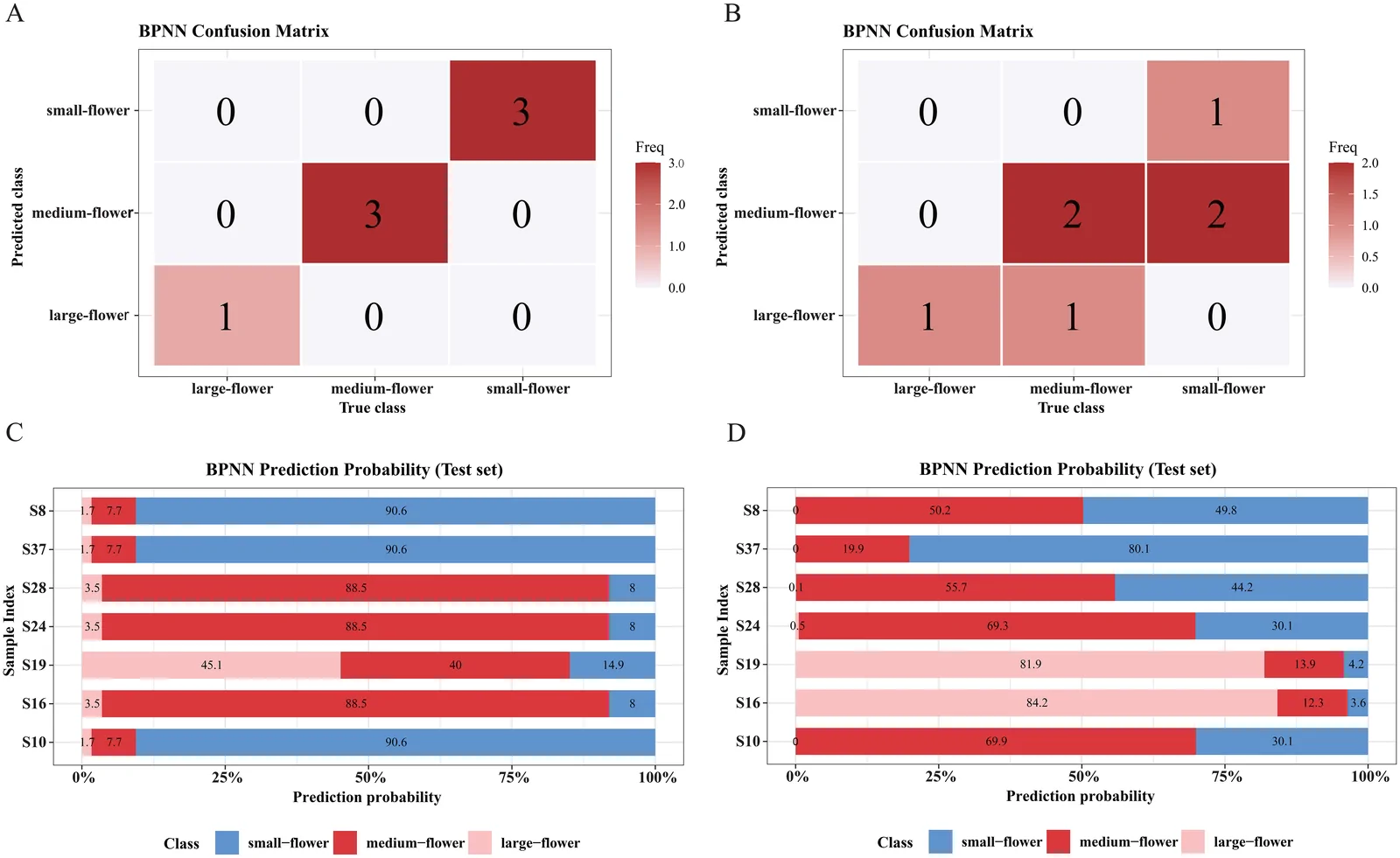
**(A)** The predicted percentage probability associated with each class of pharmacopoeia marker compounds. **(B)** Confusion matrix of pharmacopoeia marker compounds. **(C)** Confusion matrix of two candidate markers. **(D)** The predicted percentage probability associated with each class of two candidate markers.

## 4 Discussion

Random forest regression analysis in the present study suggested that commercial specification represents an important factor shaping the contents of evodiamine, rutaecarpine and limonin. Generalized linear models were further applied to cross-validate these random forest outputs. For the total content of evodiamine and rutaecarpine, maturity stage exhibited the strongest independent association (Wald *χ²*  = 119.182). Due to sample-size limitations, all second-order interaction terms were statistically non-significant (*P* ≥ 0.561). Given the evident confounding association between commercial specification and maturity stage (Cramér’s *V*  =  0.577), stratified analyses were performed. The covariation pattern between commercial specifications and maturity stages (large-flower samples predominantly collected at mature stages) partially reflects the biological basis of commercial specification for Evodiae Fructus. Since fruit development intrinsically determines fruit size, these two variables cannot be fully disentangled at both conceptual and sampling levels. The combined contents of evodiamine and rutaecarpine increased markedly along fruit development, which is consistent with previous reports regarding the developmental accumulation pattern of indole alkaloids (Sugimura et al., 2025). For limonin content, commercial specification showed the strongest independent association (Wald *χ²* = 27.815), together with a modulating effect from geographical origin (Wald *χ²* =  8.98, *P* = 0.011). This interaction indicates that the influence of commercial specification on limonin is not universally consistent across geographical backgrounds (Zhang et al., 2021; Zhao et al., 2008). According to pharmacopoeia requirements (National Pharmacopoeia Commission, 2025), medicinal Evodiae Fructus should be harvested at the pre-mature stage. Nevertheless, distinct commercial specifications were still observed within pre-mature samples in our dataset. Therefore, both commercial specification and geographical origin should be considered during quality-control practice. Considering the background of geo-authentic herbal materials (Standing Committee of the National People’s Congress, 2016), the present work mainly focused on quality evaluation related to commercial specification.

Although comparative BPNN classification models constructed with pharmacopoeia indicators and two sets of candidate markers yielded 100 % accuracy using all pre-mature samples, such high predictive performance is likely attributable to over-fitting arising from the limited sample size. Notably, these two core candidate quinolone alkaloid metabolic markers lack confirmation by authentic reference standards; their unambiguous structural elucidation and absolute quantification remain to be addressed in future work. Another important limitation of the present study is the lack of an independent external validation cohort. Owing to the restricted sample availability, all performance metrics were obtained from internal cross-validation, which generally yields over-optimistic results compared with external validation. Accordingly, the supervised models including the BPNN should be interpreted as exploratory rather than confirmatory. Before practical deployment, their predictive capacity needs further verification based on larger, independent, and preferably multi-center sample cohorts. Future investigations should expand sample collections with balanced group distributions and employ corresponding reference standards to validate and compare the quantitative performance of these candidate markers.

Moreover, because sub-samples from the same batch share the same botanical origin, geographical origin, and commercial specification but differ in maturity stage, the potential non-independence applies primarily to the maturity-stage comparisons. Nevertheless, we acknowledge that treating sub-samples from the same batch as fully independent observations may inflate the degrees of freedom and overstate the statistical significance of maturity-stage effects. This is acknowledged as a limitation of the present study, and future studies collecting multiple independent batches for each maturity stage are warranted to verify our conclusions.

## 5 Conclusions

In this study, comprehensive verification was performed by integrating multiple mathematical models and multi-dimensional variable analysis covering four crucial factors, namely commercial specification, botanical origin, maturity state and geographical origin of Evodiae Fructus. This strategy overcomes the limitations of conventional quality evaluation systems, which rely solely on single indicators or individual bioactive components. The results systematically confirmed that commercial specification serves as the core pivotal factor linking the external phenotypic characteristics and the internal accumulation of pharmacodynamic substances in Evodiae Fructus. Furthermore, two candidate markers were screened out through correlation analysis between differential metabolites and pharmacopoeia indicators. BPNN models were separately established based on conventional pharmacopoeia indicators and the two candidate markers for specification classification. Notably, the two quality markers exhibited superior classification performance. For practical applications, larger sample set should be supplemented and analyzed for confirming these preliminary results.

## Data Availability

The original contributions presented in the study are included in the article/**Supplementary Material**. Further inquiries can be directed to the corresponding author.
