# Supplementary material for: Dissection of quality variation and hierarchical quality evaluation of Evodiae Fructus under multidimensional factors
Source: Front Plant Sci. 2026 Oct 28;17:1942571. doi: 10.3389/fpls.2026.1942571 (PMC13747178; doi:10.3389/fpls.2026.1942571)
Supplement: Supplementary file 1 [file DataSheet1.docx]

**Supplementary Table 1** Cross‑tabulated distribution and association tests for the four categorical factors.

| **variable combinations** | **likelihood‑ratio χ²** | **Cramer’s V** | **P‑V** |
| --- | --- | --- | --- |
| botanical origins * geographical origins | 62.179 | 0.880 | 1.73E-18 |
| botanical origins * commercial specifications | 22.886 | 0.415 | 2.64E-4 |
| botanical origins * maturity stages | 19.678 | 0.361 | 1.28E-2 |
| geographical origins * commercial specifications | 12.288 | 0.300 | 8.35E-2 |
| geographical origins * maturity stages | 10.461 | 0.214 | 0.481 |
| commercial specifications * maturity stages | 49.05514 | 0.577 | 2.51E-7 |

**Supplementary Figure 1.**Multivariate analysis of non-targeted metabolomics data for each of the six comparison dimensions. Each row corresponds to one comparison dimension; the left panel shows the unsupervised PCA score plot, the middle panel shows the supervised OPLS-DA score plot, and the right panel shows the results of a 200-times permutation test.(A)*E. rutaecarpa* from different maturity stages.(B)Evodia Fructus from different botanical origins.(C)*E. rutaecarpa* var. *officinalis* from different commercial specifications.(D)*E. rutaecarpa* from different commercial specifications.(E)*E. rutaecarpa* var. *officinalis* from different geographical origins.(F)*E. rutaecarpa* from different geographical origins.

**Supplementary Figure 2.**Core differential metabolites were identified using Venn diagrams across six comparative dimensions.(A)*E. rutaecarpa* from different maturity stages.(B)Evodia Fructus from different botanical origins.(C)*E. rutaecarpa* var. *officinalis* from different commercial specifications.(D)*E. rutaecarpa* from different commercial specifications.(E)*E. rutaecarpa* var. *officinalis* from different geographical origins.(F)*E. rutaecarpa* from different geographical origins.

**Supplementary Figure 3** GNPS Feature-Based Molecular Networking of *E.rutaecarpa* at different maturity stages.A.Negative ion mode, B. Positive ion mode

**Supplementary Figure 4** GNPS Feature-Based Molecular Networking of Evodiae Fructus at different botanic alorigins.A.Negative ion mode, B. Positive ion mode

**Supplementary Figure 5** GNPS Feature-Based Molecular Networking of *E. rutaecarpa* var. *officinalis* at different commercial specifications.A.Negative ion mode, B. Positive ion mode

**Supplementary Figure 6** GNPS Feature-Based Molecular Networking of *E. rutaecarpa* at different commercial specifications.A.Negative ion mode, B. Positive ion mode

**Supplementary Figure 7** GNPS Feature-Based Molecular Networking of *E. rutaecarpa* var. *officinalis* at different geographical origins.A.Negative ion mode, B. Positive ion mode

**Supplementary Figure 8** GNPS Feature-Based Molecular Networking of *E. rutaecarpa* at different geographical origins.A.Negative ion mode, B. Positive ion mode

**Supplementary Table 2** Metabolite Identification Information

| **name** | **Molecularformula** | **m/z** | **RT[min]** | **Ionmode** | **M^2^m/z** | **Sourcetype** | **Spectralscore** | **MSIlevel** |
| --- | --- | --- | --- | --- | --- | --- | --- | --- |
| 5-Methoxy-N-N-dimethyltryptamine | C13 H18 N2 O | 219.14927 | 14.739 | [M+H]+1 | 174.0915;148.0759 | mzCloud;ref.[PMID: 37570816] | 76.4 | 2 |
| Dopamine | C8 H11 N O2 | 137.05991 | 1.889 | [M+H-NH3]+1 | 91.0548;119.0495;137.0598 | In-house accurate-mass list;[PMID 40632151] | NA | 3 |
| 3'-hydroxyacetophenone | C8 H8 O2 | 137.0598 | 1.889 | [M+H]+1 | 137.0598;119.0493;91.0548;65.0390 | mzCloud | 79.7 | 2 |
| 1‑Methyl‑2‑[(3E,6Z,9Z)‑3,6,9‑pentadecatrienyl]‑4(1H)‑quinolone | C25 H33 N O | 364.26347 | 39.089 | [M+H]+1 | 173.0834;186.0912 | In-house accurate-mass list;ref[PMID 12494344/27239783/28749432] | NA | 3 |
| 1‑Methyl‑2‑[(3E,6Z,9Z,12E)‑3,6,9,12‑pentadecatetraenyl]‑4(1H)‑quinolone | C25 H31 N O | 362.24787 | 37.668 | [M+H]+1 | 173.0838;186.0914 | In-house accurate-mass list;ref[PMID 12494344/27239783/28749432] | NA | 3 |
| goshuyuamide I | C19 H19 N3 O | 306.16017 | 31.693 | [M+H]+1 | 134.0602;135.0837;161.0715 | In-house accurate-mass list;ref[PMID 16681034] | NA | 2 |
| Evodiamine | C19 H17 N3 O | 304.14441 | 30.768 | [M+H]+1 | 171.0917;161.0714;134.0602;144.0809;304.1448 | Reference standard | Confirmed | 1 |
| Isoquinoline | C9 H7 N | 171.09168 | 30.765 | [M+ACN+H]+1 | 171.0917;144.0805;89.0714;77.8138 | mzCloud | 80.1 | 2 |
| Dihydrorutaecarpine | C18 H15 N3 O | 288.11454 | 28.818 | [M-H]-1 | 145.0397;169.0763;142.0651 | In-house accurate-mass list;ref[PMID 27239783] | NA | 2 |
| 3-[4-methoxy-2-[(2S,3R,4S,5S,6R)-3,4,5-trihydroxy-6-(hydroxymethyl)oxan-2-yl]oxyphenyl]propanoic acid | C16H22O9 | 357.1198 | 16.041 | [M-H]-1 | 136.0550;151.0772;  120.9403;121.0292;162.0261 | GNPS | 0.93 | 2 |
| 2-Oxo-delta3-4-5-5-trimethylcyclopentenylacetate | C10 H14 O3 | 181.0863 | 24.552 | [M-H]-1 | 141.0909;123.0803;109.0647;95.0492 | GNPS | 0.92 | 2 |
| 5D-5-O-Methyl-2-3-5/4-6-pentahydroxycyclohexanone | C7 H12 O6 | 191.05547 | 1.309 | [M-H]-1 | 191.0554；192.0589；85.0281；127.0388 | mzCloud | 91.1 | 2 |
| Pipecolic acid | C6 H11 N O2 | 130.08645 | 1.415 | [M+H]+1 | 130.0864；85.0846；84.0814；113.9874 | mzCloud | 93.3 | 2 |
| L(+)-Arginine | C6 H14 N4 O2 | 175.11908 | 1.147 | [M+H]+1 | 175.1190；158.0925；130.0976；116.0709 | mzCloud | 96.5 | 2 |
| L-Asparagine | C4 H8 N2 O3 | 133.06091 | 1.172 | [M+H]+1 | 74.0243；70.0294；87.0558；133.0608 | mzCloud | 88.1 | 2 |
| 1‑Methyl‑2‑[(1E,4Z,7Z,10E)‑1,4,7,10‑tridecatetraenyl]‑4(1H)‑quinolone | C23 H27 N O | 334.2166 | 34.798 | [M+H]+1 | 186.0914；173.0834 | In-house accurate-mass list;ref[PMID 12494344/27239783/28749432] | NA | 3 |
| 3-Methyl-quinolin-2-ol | C10 H9 N O | 160.07584 | 3.968 | [M+H]+1 | 161.0791；131.9737；132.0810 | mzCloud | 79.6 | 2 |
| 3-O-Feruloylquinic acid | C17 H20 O9 | 367.10386 | 16.885 | [M-H]-1 | 191.0556；193.0501 | mzCloud | 91.1 | 2 |
| euocarpine C | C23 H31 N O2 | 336.23224 | 36.416 | [M+H-H2O]+1 | 337.2355；334.2169；212.1071；186.0913 | In-house accurate-mass list;ref[PMID 23651559] | NA | 2 |
| 1‑Methyl‑2‑[(1E,5Z)‑1,5‑undecadienyl]‑4(1H)‑quinolone | C21 H27 N O | 310.21659 | 35.355 | [M+H]+1 | 311.2198；298.3439；256.3241；184.0763 | mzCloud | 79.1 | 3 |
| 6-methoxy-N-methyl-1，2，3，4-tetrahy- dro-β-carboline | C14 H17 N O | 216.13848 | 24.932 | [M+H]+1 | 216.1386；217.1420；173.0810；151.9395 | mzCloud | 83.6 | 2 |
| Tamarxetin | C16 H12 O7 | 317.06557 | 19.459 | [M+H]+1 | 317.0658；302.0426；153.0188 | mzCloud | 92.8 | 2 |
| Indole-3-carbinol | C9 H9 N O | 148.07578 | 4.767 | [M+H]+1 | 130.0653；103.0546；128.9514； | mzCloud | 76.7 | 2 |
| isorhamnetin-3-O-rutinoside | C28 H32 O16 | 623.1628 | 20.149 | [M-H]-1 | 623.1772；357.0565 ；317.0527；316.0747；315.0794 | mzCloud | 73.5 | 2 |
| isoferulic acid | C10H10O4 | 193.0500 | 18.913 | [M-H]-1 | 133.0293;132.0219;  134.0347 | GNPS | 0.79 | 2 |
| 3-[(2S,3R,4S,5S,6R)-4,5-dihydroxy-6-(hydroxymethyl)-3-[(2S,3R,4S,5R)-3,4,5-trihydroxyoxan-2-yl]oxyoxan-2-yl]oxy-2-(3,4-dihydroxyphenyl)-5-hydroxy-7-methoxychromen-4-one | C27H30O16 | 633.1433 | 19.449 | [M+Na]+1 | 317.0654;85.029;73.0288;  97.0284;317.2174 | GNPS | 0.92 | 2 |
| 1‑Methyl‑2‑[(Z)‑1‑undecenyl]‑4(1H)‑quinolone | C21 H29 N O | 312.23202 | 35.949 | [M+H]+1 | 173.0844；186.0924 | mzCloud | 88.3 | 2 |
| 1‑Methyl‑2‑[(Z)‑pentadec‑6‑enyl]‑4(1H)‑quinolone | C25 H37 N O | 368.29468 | 44.024 | [M+H]+1 | 173.0829；186.0917 | In-house accurate-mass list;ref[PMID 12494344/27239783/28749432] | NA | 3 |
| Limonin | C26 H30 O8 | 471.20163 | 17.693 | [M+H]+1 | 367.1959；425.1961；161.0597 | Reference standard | Confirmed | 1 |
| 1‑Methyl‑2‑[(4Z,7Z,10E)‑trideca‑4,7,10‑trienyl]‑4(1H)‑quinolone | C23 H29 N O | 336.23208 | 35.988 | [M+H]+1 | 173.0836；186.0912 | In-house accurate-mass list;ref[PMID 12494344/27239783/28749432] | NA | 3 |
| 1‑Methyl‑2‑decyl‑4(1H)‑quinolone | C20 H29 N O | 300.23221 | 36.695 | [M+H]+1 | 173.0838；186.0916；158.0606 | In-house accurate-mass list;ref[PMID 23651559] | NA | 2 |
| 1-Methyl-2-undecyl-4(1H)-quinolone | C23 H35 N O | 342.27899 | 43.265 | [M+H]+1 | 173.0830；186.0932 | In-house accurate-mass list;ref[PMID 18058680] | NA | 2 |
| 1‑Methyl‑2‑nonyl‑4(1H)‑quinolone | C19 H27 N O | 286.21635 | 34.589 | [M+H]+1 | 173.0842；186.0930 | mzCloud | 79.1 | 2 |
| 2-Tridecyl-4(1H)-quinolone | C22 H33 N O | 328.26363 | 40.31 | [M+H]+1 | 173.0841；186.0924 | mzCloud | 68.4 | 3 |
| trans-Caffeoyl‑6‑O‑D‑gluconic acid | C15 H18 O10 | 357.08323 | 7.297 | [M-H]-1 | 195.0503；163.0394 | In-house accurate-mass list; ref[PMID 25801682] | NA | 2 |
| 2-Oxindole | C8 H7 N O | 134.05985 | 29.762 | [M+H]+1 | 134.0601；135.0637 | mzCloud | 89.0 | 2 |
| Epicatechin | C15 H14 O6 | 289.07217 | 13.722 | [M-H]-1 | 289.0723；287.0566；579.1522 | mzCloud | 89.3 | 2 |
| trans‑Caffeoyl‑5‑O‑D‑gluconic acid | C15 H18 O10 | 357.08323 | 6.752 | [M-H]-1 | 195.0506；163.0397 | In-house mass list; ref[PMID 25801682] | NA | 3 |
| trans‑Caffeoyl‑4‑O‑D‑gluconic acid | C15 H18 O10 | 357.08328 | 5.081 | [M-H]-1 | 195.0506；163.0397 | In-house mass list; ref[PMID 25801682] | NA | 3 |
| 8‑Hydroxy‑4‑methoxy‑3‑(3‑methylbut‑2‑en‑1‑yl)quinolin‑2(1H)‑one | C15 H17 N O3 | 260.12791 | 27.017 | [M+H]+1 | 261.1314；204.0655；148.0391 | In-house mass list; ref[PMID: 32540378] | NA | 2 |
| L-Glutamic acid | C5 H9 N O4 | 293.09899 | 1.359 | [2M-H]-1 | 130.0499；102.0553；84.0449； | mzCloud | 88.5 | 2 |
| 3-hydroxy-1H-quinolin-4-one | C9 H7 N O2 | 162.055 | 24.321 | [M+H]+1 | 163.0752；119.0855；105.0338；117.9798 | mzCloud | 83.1 | 2 |
| 6-Prenylnaringenin | C20H20O5 | 339.1233 | 24.850845 | [M-H]-1 | 119.0518 ；133.0675；219.0680 ；151.0787；  120.0547 | GNPS | 0.78 | 2 |
| 8-isopentenylnaringenin | C20H20O5 | 341.1378 | 24.8526 | [M+H]+1 | 285.0772；165.0189；221.0819；191.0347；  147.0445 | GNPS | 0.86 | 2 |
| Rutaecarpine | C18 H13 N3 O | 288.11278 | 30.346 | [M+H]+1 | 245.7903；251.8274；63.0983 | Reference standard | Confirmed | 1 |
| 2-hydroxy-4-methoxy-3-(3’-methylbut-2’-enyl) quinolin-4-one | C15 H17 N O2 | 244.13314 | 29.915 | [M+H]+1 | 245.1366；188.0706；158.0600 | mzCloud | 75.6 | 2 |
| 2‑tridecyl‑4 (1H)‑quinolone | C22 H33 N O | 328.26363 | 40.31 | [M+H]+1 | 173.0841;186.0924 | mzCloud | 68.4 | 3 |
| 1‑Methyl‑2‑[(4Z,7Z,10E)‑trideca‑4,7,10‑trien‑1‑yl]‑4 (1H)‑quinolone | C23 H29 N O | 336.23208 | 35.988 | [M+H]+1 | 173.0836；186.0912 | In-house mass list; ref[PMID 12494344/27239783/28749432] | NA | 3 |
| 1-Methyl-2-tetradecyl-4 (1H)-quinolone | C24 H37 N O | 356.29461 | 43.122 | [M+H]+1 | 173.0835;186.0929 | In-house mass list; ref[PMID 12494344] | NA | 2 |
| 1‑Methyl‑2‑decyl‑4 (1H)‑quinolone | C20 H29 N O | 300.23221 | 36.695 | [M+H]+1 | 173.0838；186.0916；158.0606 | In-house mass list; ref[PMID 25801682] | NA | 3 |
| Isorhamnetin | C16 H12 O7 | 317.0654 | 18.278 | [M+H]+1 | 157.0436；274.0503；285.0392；302.0420；317.0655 | mzCloud | 92.8 | 2 |
| 1‑Methyl‑2‑[(Z)‑pentadec‑10‑enyl]‑4 (1H)‑quinolone | C24 H35 N O | 354.27895 | 40.297 | [M+H]+1 | 173.0842；186.0919 | In-house mass list; ref[PMID 25801682] | NA | 3 |
| Goshuyuamide Ⅱ | C19 H17 N3 O2 | 320.13929 | 20.078 | [M+H]+1 | 177.0913；149.0963 | mzCloud | 82.6 | 2 |

**Supplementary Table 3** Metabolite Identification Information

| **Indicator** | **Accuracy** | **commercial specifications** | **recall** | **precision** | **F1** |
| --- | --- | --- | --- | --- | --- |
| pharmacopoeia marker compounds | 0.833 | large-flower | 0.500 | 1.000 | 0.667 |
|  |  | medium-flower | 0.667 | 0.667 | 0.667 |
|  |  | small-flower | 1.000 | 0.667 | 0.800 |
| two candidate markers | 1.000 | large-flower | 1.000 | 1.000 | 1.000 |
|  |  | medium-flower | 1.000 | 1.000 | 1.000 |
|  |  | small-flower | 1.000 | 1.000 | 1.000 |

**References**

[1] Zhang, L., Xu, J., Guo, J., Wang, Y., et al. (2023). Elucidation of pharmacological mechanism underlying the anti‑Alzheimer's disease effects of Evodia rutaecarpa and discovery of novel lead molecules: An in silico study. Molecules, 28(15), 5846. https://doi.org/10.3390/molecules28155846.PMID: 37570816.
[2] Qian, Q., Zhuo, Z., Liao, W., et al. (2025). Multiomics analysis reveals candidate genes and pathway involved in isoquinoline alkaloids in Zanthoxylum armatum DC. fruit. Plant Molecular Biology, 115(4), 86. https://doi.org/10.1007/s11103‑025‑01605‑6. PMID: 40632151.
[3] Ko, J. S., Rho, M. C., Chung, M. Y., et al. (2002). Quinolone alkaloids, diacylglycerol acyltransferase inhibitors from the fruits of Evodia rutaecarpa. Planta Medica, 68(12), 1131‑1133. https://doi.org/10.1055/s‑2002‑36358. PMID: 12494344.
[4] Li, W., Sun, X., Liu, B., et al. (2016). Screening and identification of hepatotoxic component in Evodia rutaecarpa based on spectrum‑effect relationship and UPLC‑Q‑TOFMS. Biomedical Chromatography, 30(12), 1975‑1983. https://doi.org/10.1002/bmc.3774. PMID: 27239783.
[5] Liang, J., Chen, Y., Ren, G., et al. (2017). Screening hepatotoxic components in Euodia rutaecarpa by UHPLC‑QTOF/MS based on the spectrum‑toxicity relationship. Molecules, 22(8), 1264. https://doi.org/10.3390/molecules22081264 PMID: 28749432
[6] Choi YH, Shin EM, Kim YS, et al. (2006).Anti-inflammatory principles from the fruits of Evodia rutaecarpa and their cellular action mechanisms. Arch Pharm Res. 29(4):293-7. doi: 10.1007/BF02968573. PMID: 16681034.
[7] Wang, X. X., Zan, K., Shi, S. P., et al. (2013). Quinolone alkaloids with antibacterial and cytotoxic activities from the fruits of Evodia rutaecarpa. Fitoterapia, 89, 1‑7. https://doi.org/10.1016/j.fitote.2013.04.007.PMID: 23651559.
[8] Adams, M., Mahringer, A., Kunert, O., et al. (2007). Cytotoxicity and p‑glycoprotein modulating effects of quinolones and indoloquinazolines from the Chinese herb Evodia rutaecarpa. Planta Medica, 73(15), 1554‑1557. https://doi.org/10.1055/s‑2007‑993743.PMID: 18058680.
[9] He, Y., Li, J., Wu, H. H., et al. (2015). A new caffeoylgluconic acid derivative from the nearly ripe fruits of Evodia rutaecarpa. Natural Product Research, 29(13), 1243‑1248. https://doi.org/10.1080/14786419.2015.1024116. PMID: 25801682.
[10] Li, D. W., Zhang, M., Feng, L., et al. (2020). Alkaloids from the nearly ripe fruits of Evodia rutaecarpa and their bioactivities. Fitoterapia, 146, 104668. https://doi.org/10.1016/j.fitote.2020.104668. PMID: 32540378.
[11] Yin, Y. Y., Liu, S. S., Han, L. W., et al. (2016). Analysis of alkaloid chemical constituents and their anti‑angiogenic activities of Evodia rutaecarpa. Chinese Journal of Experimental Traditional Medical Formulae, 22(5), 45‑53. https://doi.org/10.13422/j.cnki.syfjx.2016050045.
[12] Zhao, X. M., Cheng, Y. X., Liang, C. X., et al. (2021). Chemical constituent analysis of Evodia rutaecarpa based on UPLC‑Q‑TOF‑MS/MS. Chinese Journal of Experimental Traditional Medical Formulae, 27(6), 113‑126. https://doi.org/10.13422/j.cnki.syfjx.20202051.
